# Unveiling community-level factors: a multilevel mixed-effect analysis of eye care service utilization and associated factors in Andabet, Northwest Ethiopia

**DOI:** 10.3389/fpubh.2025.1536068

**Published:** 2025-03-25

**Authors:** Zufan Alamrie Asmare, Sintayehu Simie Tsega, Tilaye Arega Moges, Getachew Yitayew Tarekegn, Dejen Gedamu Damtie, Bayih Endalew Bitew, Sisay Sitotaw Anberbr, Beminate Lemma Seifu, Fisseha Nigussie Dagnew

**Affiliations:** ^1^Department of Ophthalmology, School of Medicine and Health Science, Debre Tabor University, Debre Tabor, Ethiopia; ^2^Department of Medical Nursing, School of Nursing, College of Medicine and Health Science, University of Gondar, Gondar, Ethiopia; ^3^Department of Pharmacy, College of Health Sciences, Debre Tabor University, Debre Tabor, Ethiopia; ^4^Department of Biostatistics, College of Health Science, Debre Tabor University, Debre Tabor, Ethiopia; ^5^Department of Clinical Pharmacy, School of Pharmacy, College of Medicine and Health Sciences, University of Gondar, Gondar, Ethiopia; ^6^Department of Public Health, College of Medicine and Health Science, Samara University, Semera, Afar, Ethiopia

**Keywords:** eye care service utilization, random-effect, mixed-effect logistic regression analysis, fixed-effect, Ethiopia

## Abstract

**Background:**

Eye care service utilization (ECSU) is vital for preventing and managing visual impairment, yet its uptake remains suboptimal in many low- and middle-income countries, including Ethiopia. Visual impairment imposes significant economic and social burdens, much of which is preventable with timely screening and treatment. Despite previous studies on ECSU, gaps remain in understanding individual- and community-level factors influencing its use in specific regions. This study aimed to determine the magnitude and associated factors of eye care service utilization (ECSU) among older adults in the Andabet District, Northwest Ethiopia.

**Method:**

A community-based cross-sectional study was carried out among older adults from May 1–30, 2024 in Andabet District, Northwest Ethiopia. Multistage systematic random sampling was employed to reach 570 older adults. A multilevel mixed-effect logistic regression analysis was employed to assess both individual and community-level factors associated with ECSU. We fitted both random-effect and fixed-effect analysis. Finally, variables with *p* < 0.05 in the multivariable multilevel mixed-effect analysis were claimed to be significantly associated with ECSU.

**Result:**

In this study, the good level of ECSU was found to be 16.14% (95% CI: 13.11, 19.16). In the multilevel mixed-effect logistic regression analysis, aged ≥ 65 years (AOR = 4.59; 95% CI: 1.38, 15.21), having health insurance (AOR = 1.98, 95% CI: 1.51, 2.58), living nearer to eye care facility (AOR = 6.42, 95% CI: 1.95, 21.15), and having awareness about regular eye checkups (AOR = 1.63; 95% CI: 2.88, 9.70) were significantly associated with good level of ECSU.

**Conclusion:**

In this study, the magnitude of eye care service utilization was lower than in other studies. Age, health insurance, distance from the eye care facility, and awareness were independent determinants of ECSU. Therefore, policymakers should prioritize accessible health insurance and expand eye care facilities, especially in rural areas. Integrating routine eye exams into primary care and launching widespread awareness campaigns can promote preventive eye health. A unified, multi-sectoral strategy focused on access, integration, and education is key to enhancing ECSU.

## Introduction

Eye care service utilization (ECSU) is essential for preventing vision loss, maintaining eye health, and ensuring timely management of ocular conditions (1, 2). However, ECSU rates vary significantly worldwide due to differences in healthcare accessibility, socioeconomic conditions, and awareness, ranging from 18 to 90% (2, 3). High-income countries, such as the United States, Australia, and Canada, report utilization rates between 60 and 90%, largely due to well-established healthcare systems and strong preventive care policies (3, 4). In contrast, ECSU remains much lower in resource-limited settings, with rates of 22% in low-income countries, 24% in lower-middle-income countries, and 37% in upper-middle-income countries (5). Similarly, ECSU in Ethiopia remains low, with notable regional variations; 21.1% in Northern Ethiopia (6), 21.6% in Southern Ethiopia (7), and 32.98% in Central Ethiopia (8), underscoring the need to identify key barriers and improve service delivery across different regions.

The consequences of low ECSU are far-reaching, fueling the global rise in visual impairment and blindness (9, 10). In Ethiopia, vision loss imposes a significant economic strain, with both direct costs like medical expenses and indirect costs, such as lost productivity (11, 12). Nearly one in 10 adults over 60 suffer from visual impairment, much of which could be prevented with routine eye exams (13). Despite strong recommendations from organizations like the American Academy of Ophthalmology, financial barriers, limited eye care services, and lack of awareness continue to hinder ECSU in many low- and middle-income countries (14, 15). As a result, millions remain at risk. Globally, 36 million people are blind, 217 million face moderate or severe visual impairment, and 188 million have mild vision loss (16). Alarmingly, nearly 40% of blindness cases could have been prevented with timely screening and treatment (17).

Despite extensive search on ECSU in Ethiopia, most studies have focused on individual-level factors such as age, income, and health insurance, with limited attention to structural and environmental barriers at the community level. In rural districts like Andabet, where healthcare infrastructure is sparse and long distances to eye care facilities pose significant challenges (18–20), understanding how these factors influence ECSU is critical. This study bridges that gap by incorporating both individual and community-level factors through multilevel mixed-effect analysis, providing a more thorough understanding of access disparities. Unique challenges in the district including limited access to transportation and healthcare infrastructure, compounded by cultural beliefs and traditional practices underscore the urgent need to investigate local cultural influences on ECSU and develop tailored interventions to address them. By emphasizing these localized challenges in line with the World Health Organization's (WHO) Universal Eye Health Action Plan, the study will advocate for decentralized eye care solutions and offer actionable, context-specific recommendations for Ethiopian policymakers and healthcare planners. More generally, it will provide a scalable model for other low-resource settings, helping to reduce preventable vision impairment and promote universal eye health.

## Methods

### Ethics statement

This study was conducted following the principles of the Declaration of Helsinki and received ethical approval from the Institutional Review Board of Debre Tabor University, Health Science College (Reference Number: 1099). Additionally, permission was granted by the Andabet District administrative office. Written informed consent was obtained from all voluntary participants, who were reassured that their participation posed no risk of harm. To safeguard confidentiality, personal identifiers were omitted, and unique codes were used to anonymize the data.

### Study design and settings

This community-based cross-sectional study was conducted among older adults aged 40 years and above from May 1–30, 2024, in Andabet District. Located 150 km from Bahir Dar, the capital of the Amhara National Regional State, and 717 km from Addis Ababa, Ethiopia's capital, Andabet spans a vast geographical area with a notably high population density. According to the 2021 regional census, the district has an estimated total population of 153,694, distributed across 35,875 households within 26 kebeles. The district is served by a primary healthcare center and two health posts, providing essential medical services to the community.

### Study population and eligibility criteria

The source population for this study included all older adults aged 40 years and above residing in the Andabet District. We included individuals who were 40 years or older and had lived in the study area for at least 6 months.

### Sample size determination and sampling procedure

The required sample size was determined using the single population proportion formula, drawing on findings from a similar study conducted in Hawassa, Ethiopia, which reported a prevalence of 23.8% (9). Accounting for a 95% confidence level, a ± 5% margin of error, a design effect of 2.0, and an additional 10% to accommodate potential non-responses, the final sample size for this study was determined to be 612.

Multistage sampling was used throughout the sampling process. First, six kebeles were randomly chosen from a list of 26 provided by the Andabet District Administration Bureau using a simple random sampling method. To ensure representativeness, the sample size for each selected kebele was determined by population proportional allocation. Next, a systematic random sampling technique with an interval of 8 was applied to select households. The first household was chosen using a lottery method, followed by the selection of every Kth household thereafter (K = N/n, where *N* = 4,881 and *n* = 612). Before initiating the sampling, a pen-spinning technique was used to determine the starting point. In households with multiple eligible individuals, one participant was randomly selected using a lottery method.

### Operational definition

#### ECSU

An individual was classified as having a good level of ECSU if they reported visiting an eye care facility for a checkup or examination at least once within the past 2 years (8).

#### Older adults

Individuals aged 40 years and above. They were selected because they face a higher risk of vision impairment and ocular diseases. They are also more likely to require eye care services, yet often encounter significant barriers to access, especially in low-resource settings like Andabet (8).

### Data collection tools and procedure

The data collection tool was developed based on a comprehensive review of the existing literature. A pretested structured questionnaire was used to gather data through interviewer-administered surveys, covering sociodemographic details (e.g., kebele, age, sex, residence, religion, education, and occupation) and medical factors such as health insurance status, diabetes, hypertension, proximity to eye care services, use of traditional eye medicine, and ECSU. Four data collectors, all BSc Optometrists, conducted the interview under the supervision of an MSc Optometrist. The collected data were then organized in an Excel format (Supplementary Table S1).

### Data quality control

To maintain consistency, the data collection tool was first developed in English, translated into the local language (Amharic), and then back-translated into English. Subsequently, data collectors and the supervisor underwent 2 days of training on data collection procedures. A pre-test was conducted on 5% of the total sample size in a different kebele that was not part of the main study. Based on the pre-test findings, the questions were revised and refined. Supervisors and investigators then reviewed the data to ensure coherence, accuracy, and clarity.

### Data processing and statistical analysis

Data entry was performed using Epi-Data version 4.6, while STATA 16 was used for data cleaning, coding, and analysis. To identify factors associated with ECSU, a multilevel mixed-effects logistic regression, accounting for the hierarchical nature of the data, where older adults were grouped within clusters. Multilevel analysis is particularly suited for such nested data structures, as it addresses key assumptions that standard logistic regression does not. Unlike standard models, which assume independence among observations, multilevel analysis accounts for clustering, where individuals within the same group may share similar characteristics. It also incorporates random effects, allowing for variability both within and between clusters, whereas standard logistic regression assumes fixed effects. Additionally, multilevel models recognize that variance may differ across groups, addressing the equal variance assumption of standard models. By considering these factors, multilevel analysis improves the accuracy of statistical inferences when dealing with hierarchical data.

Initially, a bivariable multilevel logistic regression analysis was conducted, and variables with a *p* < 0.20 were selected for the multivariable multilevel analysis. During the multilevel binary logistic regression, both random and fixed-effect models were fitted. The intraclass correlation coefficient (ICC), a random effect parameter, used to measure the degree of variability in ECSU across clusters. An ICC >10% indicated the importance of considering cluster-level variability, thereby making multilevel analysis absolutely crucial. Additionally, proportional change in variance (PCV) and median odds ratio (MOR) were assessed. Multicollinearity was tested using the variance inflation factor (VIF), with all independent variables showing a VIF of less than five and a mean VIF of 1.85, indicating no significant multicollinearity among the variables.

In the fixed-effect analysis, four models were fitted: model 1 (without explanatory variables), Model 2 (including only individual-level factors), Model 3 (including community-level factors), and Model 4 (incorporating both individual and community-level factors). Of these models, Model 4 was identified as the best-fit model due to its lowest deviance and highest PCV. The adjusted odds ratio (AOR) with its 95% CI was presented for each fitted model, but interpretations were based solely on the final, best-fit model. Variables with a *p* < 0.05 in the multivariable multilevel analysis were considered significantly associated with ECSU.

## Result

### Socio-demographic characteristics of study participants

A total of 570 adults aged 40 and above participated in this study, with a response rate of 93.14%. More than half (52.11%) were between 55 and 64 years old, with a mean age of 59 ± 5. Among the participants, 286 (50.18%) resided in rural areas, and 46.67% were engaged in farming. Regarding family monthly income, approximately three-quarters (75.43%) earned <4,000 ETB (Table 1).

**Table 1:** Socio-demographic characteristics of older adults in Andabet, Northwest Ethiopia: a multi-level mixed-effect analysis (*n* = 570).

**Variables**	**Category**	**Frequency**	**Percentage**	**Mean (SD)**
Age (in years)	40–54	71	12.46	59 (±5)
	55–64	297	52.11	
	65 yrs and above	202.	35.44	
Sex	Male	291	51.05	N/A
	Female	279	48.95	
Residence	Rural	286	50.18	N/A
	Urban	284	49.82	
Religion	Orthodox	527	92.46	N/A
	Muslim	43	7.54	
Educational level	Cannot write and read	229	40.18	N/A
	Write and read	96	16.84	
	Primary education	146	25.61	
	Secondary education	69	12.11	
	College and above	30	5.26	
Occupation	Farmer	266	46.67	N/A
	Housewife	162	28.42	
	Government employee	35	6.14	
	Merchant	72	12.63	
	Daily laborer	35	6.14	
Marital status	Currently in union	427	74.91	N/A
	Currently not in union	143	25.09	
Family monthly income in ETB	500–2,000	282	49.47	2709.78 (±1683.57)
	2,001–4,000	148	25.96	
	4,001–5,999	92	16.14	
	≥6,000	48	8.42	

SD, standard deviation; N/A, not available for non-continuous variables.

### Medical and related characteristics of study participants

A total of 254 participants (44.64%) had a history of eye diseases. Among them, nearly half (47.25%) reported that their eye condition affected their daily activities. However, the majority (89.12%) were unaware of the importance of regular eye checkups, and approximately half (48.07%) lived far from healthcare facilities (Table 2).

**Table 2:** Medical and related characteristics of older adults in Andabet, northwest Ethiopia: a multi-level mixed-effect analysis (*n* = 570).

**Variables**	**Category**	**Frequency**	**Percentage**
Health insurance	Yes	292	51.23
	No	278	48.77
DM	Yes	355	62.28
	No	215	37.72
Hypertension	Yes	456	80.00
	No	114	20.00
Distance to the nearest eye care facility	≥30 min	274	48.07
	<30 min	296	51.93
Had escort	Yes	115	20.18
	No	455	79.82
Awareness about regular eye checkup	Yes	62	10.88
	No	508	89.12
History of eye disease	Yes	254	44.64
	No	316	55.36
Eye problem affect daily activity (*n* = 254)	Yes	120	47.25
	No	134	52.75
Reason for not visiting eye care service	Due to financial problem	266	55.64
	I don't know the place	162	33.9
	I used traditional medicine	50	10.46

### Eye care service utilization

This study revealed that 16.14% (95% CI: 13.11, 19.16) of participants had a good level of ECSU, while the remaining 83.86% had a poor level.

### Model fitness

In the random-effect analysis, Model 1 showed that around 70.3% of the total variation in ECSU was observed at the cluster level and attributed to community-level factors. Moreover, Model 1 had the highest median odds ratio (MOR) of 14.15, indicating that individuals in a kebele with a higher risk of poor ECSU were 14.15 times more likely to experience poor ECSU compared to those in a lower-risk kebele. Additionally, the final model (Model 4) had the highest proportional change in variance (PCV) at 88%, demonstrating that both individual and community-level factors explained 88% of the variation in ECSU across communities. Model fitness was assessed using deviance, and Model 4, which exhibited the lowest deviance, was determined to be the best-fitted model (Table 3).

**Table 3:** Random effect and model fitness of eye care service utilization and associated factors in Andabet district, northwest Ethiopia: a multi-level mixed-effect analysis (*n* = 570).

**Parameter**	**Model 1**	**Model 2**	**Model 3**	**Model 4**
Log likelihood	−229.30	−185.15	−206.9	−180.09
MOR	14.15	13.32	8.16	2.46
PCV	Ref.	0.042	0.36	0.88
ICC	0.703	0.694	0.602	0.216
Deviance	458.6	370.3	413.8	360.18

MOR, median odds ratio; PCV, proportional change in variance; ICC, intraclass correlation coefficient.

### Factors associated with eye care service utilization

In multivariable multilevel logistic regression analysis, where both the individual and community level factors were fitted simultaneously; age, health insurance, distance to the nearest eye care facility, and awareness about regular eye check-ups were significantly associated with good level of ECSU.

Individuals in the age group of 65 and above were 4.59 times more likely to utilize eye care services (AOR = 4.59; 95% CI: 1.38, 15.21) as compared to individuals in the age group 40–54 years. Similarly, Participants who had health insurance were 1.98 times more likely to utilize eye care services (AOR = 1.98, 95% CI: 1.51, 2.58) as compared to their counterparts. Regarding distance to the nearest eye care facility, Participants living nearer to the eye care facility were 6.42 times more likely to utilize eye care service (AOR = 6.42, 95% CI: 1.95, 21.15) as compared to their counterparts. In the same manner, the odds of using eye care service from participants who had awareness about regular eye checkups were 1.63 (AOR = 1.63; 95% CI: 2.88, 9.70) times higher than their counterparts (Table 4).

**Table 4:** Multi-level mixed-effect logistic regression analysis for factors associated with eye care service utilization among older adults in Andabet district, Northwest Ethiopia (*n* = 570).

**Variables**	**Model 1**	**Model 2 AOR 95% (CI)**	**Model 3 AOR 95% (CI)**	**Model 4 AOR 95% (CI)**	***p*-value for model 4 (best fitted model)**
**Age (in years)**					
40–54		1.00		1.00
55–64		2.35 (0.76, 7.30)		1.95 (0.62, 6.09)	0.76
65 yrs and above		4.68 (1.41, 15.48)^*^		4.59 (1.38, 15.21)^*^	0.03
**Sex**					
Female		1.00		1.00
Male		0.61 (0.34, 1.06)		0.65 (0.36, 1.15)	0.85
Residence		0.85 (0.46, 1.56)		0.89 (0.50, 1.61)	0.96
Rural		1.00		1.00
Urban			2.20 (0.57, 8.39)	0.28 (0.02, 3.62)	0.75
**Marital status**					
Currently in union		1.00		1.00
Currently not in union		1.48 (0.69, 3.18)		1.29 (0.59, 2.81)	0.83
**Occupation**					
Housewife		1.00		1.00
Farmer		2.27 (0.97, 5.32)		1.97 (0.83, 4.65)	0.95
Government employed		2.47 (0.55, 10.97)		1.95 (0.41, 9.18)	0.92
Merchant		1.98 (0.65, 5.99)		2.07 (0.68, 6.25)	0.80
Daily laborer		0.75 (0.08, 6.45)		0.72 (0.08, 6.24)	0.45
**Health insurance**					
Yes				1.98 (1.51, 2.58)^**^	0.001
No		1.00		1.00
**Distance to the nearest eye care facility**					
<30 min walking time			10.56 (3.96, 28.12) ^**^	6.42 (1.95, 21.15)^**^	0.003
≥30 min walking time			1.00	1.00
**Hypertension**					
Yes		0.51 (0.26, 1.00)		0.59 (0.29, 1.20)	0.86
No		1.00		1.00
**DM**					
Yes		0.76 (0.41, 1.41)		0.89 (0.47, 1.69)	0.74
No		1.00		1.00
**Aware about regular eye checkups**					
Yes		1.95 (0.44, 0.86)^*^		1.63 (0.34, 7.81)^*^	0.03
No		1.00		1.00

^**^P < 0.01 and ^*^P < 0.05.

## Discussion

This study found the magnitude of ECSU to be 16.14%, a rate consistent with studies conducted in Northwest Ethiopia (6), Gamo Gofa [Southern Ethiopia; (7)], and Yobe, Nigeria (21). This similarity may stem from comparable healthcare access and sociodemographic factors, such as education level and income (22). Specifically, in these Ethiopian local regions, lower education levels and economic constraints may limit awareness and affordability of eye care services, resulting in similar utilization patterns (23). However, this magnitude of ECSU was found to be lower compared to different studies conducted in Hawassa (9), Debre Birhan (8), Gondar (24), Ghana (25), Edo state of Nigeria (9), Abuja Nigeria (26), Limpopo province of South Africa (26), United States (27) and Australia (28). Several factors may explain this difference, including variations in healthcare infrastructure, socio-demographic characteristics, and cultural influences (29). Urban Ethiopian areas like Hawassa, Debre Birhan, and Gondar typically have better-equipped healthcare facilities, a higher concentration of trained eye care professionals, and more specialized services compared to rural areas, which likely contributes to higher utilization rates (30). Additionally, the findings from studies conducted outside Ethiopia may be influenced by socio-demographic factors such as higher income levels, better socioeconomic conditions, and more established health systems, all of which contributed to greater healthcare utilization. In contrast, economically disadvantaged populations, particularly in rural Ethiopia like Andabet, often face challenges such as high out-of-pocket costs, limited transportation access, and competing health priorities, which deter them from seeking eye care services (31). Cultural factors also play a role, as countries like United States, Australia, Ghana and Nigeria may benefit from more robust community outreach programs and public awareness campaigns about eye health, encouraging earlier detection and treatment, while some Ethiopian communities may rely on traditional healing practices or have lower awareness of the importance of eye care, leading to lower utilization rates until vision problems become more severe (32). All these aspects highlight the vital role of healthcare accessibility, socioeconomic conditions, and cultural awareness in shaping ECSU rates, emphasizing the need for targeted interventions to improve ECSU, particularly in underserved regions.

In the multivariable multi-level logistic regression; age, health insurance, distance to the nearest eye care facility, and awareness about regular eye check-ups were significantly associated with ECSU. Consistent with different studies conducted in Abuja, Nigeria, South Korea, Tehran, Australia, and America (21, 26, 28, 33–35), Individuals in the age group of 65 and above had higher odds of ECSU as compared to their counterparts. This might be because, as people age, eye conditions like cataracts, macular degeneration, glaucoma, and presbyopia become more prevalent and pronounced. Additionally, older adults are more susceptible to chronic health conditions such as hypertension and diabetes, which can have harmful effects on their vision. As a result, regular eye exams are essential not only for early detection but also for managing these age-related eye health concerns effectively (36).

Health insurance is a factor for ECSU in which, Participants who have health insurance had higher odds of ECSU as compared to their counterparts. This finding is supported by a study done in the United States (37). This might be because health insurance makes eye care services more accessible by reducing financial barriers, covering routine exams, prescription eyewear, treatments, and emergency care. With these services within reach, individuals are more likely to prioritize their eye health and seek timely care when needed.

In this study, participants living near to eye care facility had higher odds of ECSU as compared to their counterparts. This finding was supported by a study done in western Kenya (38). This could be because the proximity of eye care facilities enhances convenience, reduces transportation obstacles, and fosters greater awareness and trust. All of these factors contribute to higher utilization rates among individuals living closer to these facilities.

Consistent with studies done in Hawassa and Australia (9, 28), participants who were aware of regular eye checkups had higher odds of ECSU as compared to their counterparts. The reason might be due to awareness fosters a proactive approach to eye health. Educated individuals understand the importance of routine exams and the early detection of potential issues. This knowledge encourages them to seek timely care, leading to increased utilization.

Unlike previous studies conducted in urban or high-income settings with well-established healthcare infrastructures, our study provides novel insights into eye care service utilization in a low-resource rural Ethiopian context, where unique structural and socioeconomic barriers exist. In Andabet, limited eye care facilities, long travel distances, low health insurance coverage, and poor awareness about regular eye check-ups significantly hinder access to care. Ethiopia's community-based health insurance (CBHI) is still in its early stages, and its role in improving eye care access remains underexplored. Additionally, the cultural perception of eye diseases, lower literacy rates, and delayed healthcare-seeking behavior in this setting differ from populations studied in high-income countries, where preventive eye care is more common. Also, unlike previous rural-based Ethiopian studies that use the traditional regression model, our study applies a multi-level mixed-effect analysis, which accounts for both individual-level and community-level factors influencing eye care utilization. This approach captures variations at different levels (household, community, healthcare system), providing a more comprehensive understanding of access barriers. The multi-level approach also helps to reduce cluster-level confounding bias, intraclass correlation bias, and aggregation bias, ensuring more reliable and robust findings.

### Strengths and limitations of the study

There were strengths and foibles in this study. To begin with the strength, the study exploits multi-level mixed effect modeling taking into account the clustering effect to draw valid inferences and conclusions. Moreover, to ascertain representativeness, the study uses an adequate sample size. However, this study had limitations: As it's a cross-sectional study, it may not exhibit a true temporal relationship between the outcome and the independent variables. Besides Social desirability bias might be introduced while assessing sensitive variables. Future research should focus on identifying additional barriers to eye care service utilization in Ethiopia, exploring the impact of socioeconomic factors, and evaluating the effectiveness of targeted interventions aimed at improving access to and awareness of eye care services.

## Conclusion

In this study, the magnitude of ECSU was lower than in other studies. Age, health insurance, distance to the nearest eye care facility, and awareness about regular eye check-ups were independent determinants of ECSU. These findings underscore the urgent need for policy-driven interventions to improve access and ensure fair distribution of eye care services. Therefore, policymakers should focus on strengthening health insurance enrollment and ensuring its accessibility for all, particularly in rural communities. They must also invest in strategically expanding eye care facilities to reduce geographic disparities and integrate routine eye examinations into primary healthcare services. Additionally, large-scale public awareness campaigns are crucial to foster a culture of preventive eye care. A well-coordinated, multi-sectoral policy approach centered on accessibility, integration, and education is imperative to improving eye care utilization and reducing the burden of vision impairment.

## Data Availability

The datasets presented in this study can be found in online repositories. The names of the repository/repositories and accession number(s) can be found in the article/Supplementary material.

## Supplementary material

Supplementary Table S1 Data set.

## References

[1] Carrasquillo O. Health care utilization. Encycl Behav Med. (2013) 22:1001–2.

[2] Berhane Y Worku A Bejiga A Adamu L Alemayehu W Bedri A. Prevalence and causes of blindness and low vision in Ethiopia. Ethiop J Health Dev. (2007) 21:204–10. 10.4314/ejhd.v21i3.10050

[3] Ehrlich JR Ndukwe T Solway E Woodward MA Singer DC Newman-Casey PA. Self-reported eye care use among US adults aged 50 to 80 years. JAMA Ophthalmol. (2019) 137:1061–6. 10.1001/jamaophthalmol.2019.1927 31219510 PMC6587143

[4] Warburg M. Visual impairment in adult people with intellectual disability: literature review. J Intellect Disabil Res. (2001) 45:424–38. 10.1046/j.1365-2788.2001.00348.x 11679048

[5] Vela C Samson E Zunzunegui MV Haddad S Aubin M-J Freeman EE. Eye care utilization by older adults in low, middle, and high income countries. BMC Ophthalmol. (2012) 12:1–7. 10.1186/1471-2415-12-5 22471351 PMC3378437

[6] Assaye AK Tegegn MT Belete GT. Eye care utilization among older subjects with visual impairment in Northwest Ethiopia. J Ophthalmic Vis Res. (2023) 18:306. 10.18502/jovr.v18i3.13779 37600912 PMC10432933

[7] Alemu T Seyum D Gebre M Sisay A Sorato MM. Level of eye healthcare utilisation and associated factors in Gamo and Gofa Zones, Southern Ethiopia: a community-based, cross-sectional study. BMJ Open. (2024) 14:e082612. 10.1136/bmjopen-2023-082612 39414302 PMC11481142

[8] Bekele MM Shumye AF Tegegn MT. Eye care service utilization and associated factors among adults in Debre Berhan Town, North Shewa, Ethiopia, 2023. Front Public Health. (2024) 12:1440357. 10.3389/fpubh.2024.1440357 39376997 PMC11457573

[9] Morka ED Yibekal BT Tegegne MM. Eye care service utilization and associated factors among older adults in Hawassa city, South Ethiopia. PLoS ONE. (2020) 15:e0231616. 10.1371/journal.pone.0231616 32298344 PMC7162464

[10] Beauchamp GR Ellepola C Beauchamp CL. Evidence-based medicine: the value of vision screening. Am Orthopt J. (2010) 60:23–7. 10.3368/aoj.60.1.23 21061880

[11] Marques AP Ramke J Cairns J Butt T Zhang JH Jones I. The economics of vision impairment and its leading causes: a systematic review. EClinicalMedicine. (2022) 46:101354. 10.1016/j.eclinm.2022.101354 35340626 PMC8943414

[12] Vanderpuye I Nyame I Okai M-P. Challenges inherent in the academic endeavours of students with visual impairment. Int J Inclus Educ. (2024) 28:1954–67. 10.1080/13603116.2022.2036830

[13] Chuck RS Dunn SP Flaxel CJ Gedde SJ Mah FS Miller KM. Comprehensive adult medical eye evaluation preferred practice pattern^®^. Ophthalmology. (2021) 128:P1–P29. 10.1016/j.ophtha.2020.10.024 34933742

[14] Yusufu M Bukhari J Yu X Lin TP Lam DS Wang N. Challenges in eye care in the Asia-Pacific region. Asia Pac J Ophthalmol. (2021) 10:423–9. 10.1097/APO.0000000000000391 34516436

[15] Sengo DB Marraca NA Muaprato AM García-Sanjuan S Caballero P López-Izquierdo I. Barriers to accessing eye health services in suburban communities in Nampula, Mozambique. Int J Environ Res Public Health. (2022) 19:3916. 10.3390/ijerph19073916 35409600 PMC8997994

[16] Bourne RR Flaxman SR Braithwaite T Cicinelli MV Das A Jonas JB. Magnitude, temporal trends, and projections of the global prevalence of blindness and distance and near vision impairment: a systematic review and meta-analysis. Lancet Glob Health. (2017) 5:e888–e97. 10.1016/S2214-109X(17)30293-0 28779882

[17] Ocansey S Kumi-Kyereme A Awusabo-Asare K Ilechie AA Boadi-Kusi SB Abraham CH. Utilization of eye care services among Ghanaian elderly population: evidence from a peri-urban community. Am Orthopt J. (2013) 2:123. 10.9734/OR/2013/5543

[18] Yirga GK Bantie B Hiruy EG Baye AA Kerebeh G Shiferaw K. Health checkup practice and its associated factors among adults in South Gondar zone Ethiopia. Sci Rep. (2024) 14:21237. 10.1038/s41598-024-69921-3 39261526 PMC11390744

[19] Kifle M Mbarika VW Datta P. Telemedicine in sub-Saharan Africa: the case of teleophthalmology and eye care in Ethiopia. J Am Soc Inf Sci Technol. (2006) 57:1383–93. 10.1002/asi.20448

[20] Hamed MM Masoud MA. An exploratory assessment of self-reported satisfaction with infrastructure and out-of-home activities for people with vision impairments. Vision. (2023) 7:58. 10.3390/vision7030058 37756132 PMC10535916

[21] Olusanya BA Ashaye AO Owoaje ET Baiyeroju AM Ajayi BG. Determinants of utilization of eye care services in a rural adult population of a developing country. Middle East Afr J Ophthalmol. (2016) 23:96. 10.4103/0974-9233.164621 26957847 PMC4759912

[22] Hwang J Rudnisky C Bowen S Johnson JA. Socioeconomic factors associated with visual impairment and ophthalmic care utilization in patients with type II diabetes. Can J Ophthalmol. (2015) 50:119–26. 10.1016/j.jcjo.2014.11.014 25863851

[23] Schaumberg DA Christen WG Glynn RJ Buring JE. Demographic predictors of eye care utilization among women. Med Care. (2000) 38:638–46. 10.1097/00005650-200006000-00005 10843311

[24] Adimassu NF Asefa N Anibesei D Belete G. Poor eye care service utilization among adults in Gondar City, Northwest Ethiopia. EC Ophthal. (2018) 9:647–57.

[25] Ilechie AA Otchere H Darko-Takyi C Halladay AC. Access to and utilization of eye care services in Ghana. PLoS ONE. (2013) 21:4.

[26] Ibeneche H Ekpenyong B Ebri A. Barriers to accessing eye care services in the federal capital territory, Abuja, Nigeria. J Niger Optom Assoc. (2018) 20:64–9. 10.13140/RG.2.2.24493.74724

[27] Wilson FA Stimpson JP Wang Y. Inconsistencies exist in national estimates of eye care services utilization in the United States. J Ophthalmol. (2015) 2015:435606. 10.1155/2015/435606 26346484 PMC4546761

[28] Foreman J Xie J Keel S Taylor HR Dirani M. Utilization of eye health-care services in Australia: the national eye health survey. Clin Experiment Ophthalmol. (2018) 46:213–21. 10.1111/ceo.13035 28793183

[29] Owusu-Afriyie B Peter N Ivihi F Kopil I Gende T. Barriers to the uptake of eye care services: a cross-sectional survey from rural and urban communities. PLoS ONE. (2024) 19:e0308294. 10.1371/journal.pone.0308294 39146331 PMC11326634

[30] Devi GV Anand B. A review of the determinants that influence the use of eye care servic. Journal of Positive School Psychology. (2022) 6:2992–3000.

[31] Melese M Alemayehu W Friedlander E Courtright P. Indirect costs associated with accessing eye care services as a barrier to service use in Ethiopia. Trop Med Int Health. (2004) 9:426–31. 10.1111/j.1365-3156.2004.01205.x 14996373

[32] Solomon SD Shoge RY Ervin AM Contreras M Harewood J Aguwa UT. Improving access to eye care: a systematic review of the literature. Ophthalmology. (2022) 129:e114–e26. 10.1016/j.ophtha.2022.07.012 36058739

[33] Park YS Heo H Ye BJ Suh Y-W Kim S-H Park SH. Prevalence and factors associated with the use of eye care services in South Korea: Korea national health and nutrition examination survey 2010–2012. Korean J Ophthalmol. (2017) 31:58–70. 10.3341/kjo.2017.31.1.58 28243025 PMC5327176

[34] Fotouhi A Hashemi H Mohammad K. Eye care utilization patterns in Tehran population: a population based cross-sectional study. BMC Ophthalmol. (2006) 6:1–5. 10.1186/1471-2415-6-4 16423308 PMC1382253

[35] Jiang X Varma R Torres M Hsu C McKean-Cowdin R Group CAES. Self-reported use of eye care among adult Chinese Americans: the Chinese American eye study. Clin Exp Ophthalmol. (2017) 176:183–93. 10.1016/j.ajo.2017.01.018 28161048 PMC5404385

[36] Kado DM Huang MH Karlamangla AS Barrett-Connor E Greendale GA. Hyperkyphotic posture predicts mortality in older community-dwelling men and women: a prospective study. J Am Geriatr Soc. (2004) 52:1662–7. 10.1111/j.1532-5415.2004.52458.x 15450042

[37] Wagner LD Rein DB. Attributes associated with eye care use in the United States: a meta-analysis. Ophthalmology. (2013) 120:1497–501. 10.1016/j.ophtha.2012.12.030 23511113 PMC3690143

[38] Rono MMed HK Macleod D Bastawrous A Wanjala E Gichangi M Burton MJ. Utilization of secondary eye care services in western Kenya. Clin Exp Ophthalmol. (2019) 16:3371. 10.3390/ijerph16183371 31547252 PMC6766006

